# 4-(4-Hy­droxy­methyl-1*H*-1,2,3-triazol-1-yl)benzoic acid

**DOI:** 10.1107/S1600536811022409

**Published:** 2011-06-18

**Authors:** Dayang Hazwani Abang Ishak, Hairul Anuar Tajuddin, Zanariah Abdullah, Siti Nadiah Abd Halim, Edward R. T. Tiekink

**Affiliations:** a Department of Chemistry, University of Malaya, 50603 Kuala Lumpur, Malaysia

## Abstract

In the title compound, C_10_H_9_N_3_O_3_, there is a small twist between the benzene and triazole rings [dihedral angle = 6.32 (7)°]; the carb­oxy­lic acid residue is almost coplanar with the benzene ring to which it is attached [O—C—C—C torsion angle = 1.49 (19)°]. The main deviation from coplanarity of the non-H atoms is found for the hy­droxy group which is almost perpendicular to the remaining atoms [N—C—C—O torsion angle = −75.46 (16)°]. In the crystal, the presence of O—H⋯O (between carboxyl groups) and O—H⋯N (between the hy­droxy group and the triazole ring) hydrogen bonds leads to supra­molecular chains along [03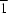]. The chains are connected into sheets *via* C—H⋯O(hy­droxy) inter­actions.

## Related literature

For background to the fluorescence potential, see: McCaroll & Wandruzska (1997 ▶). For synthetic protocols, see: Rostovtsev *et al.* (2002 ▶); Ryu & Zhao (2005 ▶); Himo *et al.* (2005 ▶). For additional geometric analysis, see: Spek (2009 ▶).
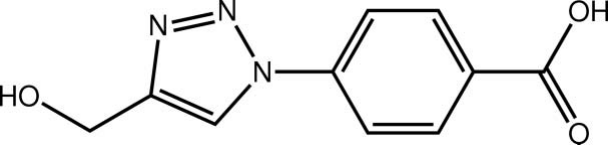


## Experimental

### 

#### Crystal data

C_10_H_9_N_3_O_3_
*M* *_r_* = 219.20
Triclinic, 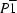

*a* = 5.4641 (7) Å
*b* = 6.6596 (8) Å
*c* = 13.1898 (16) Å
α = 88.828 (2)°
β = 83.577 (2)°
γ = 75.828 (2)°
*V* = 462.42 (10) Å^3^
*Z* = 2
Mo *K*α radiation
μ = 0.12 mm^−1^
*T* = 100 K
0.20 × 0.20 × 0.18 mm

#### Data collection

Bruker SMART APEX CCD diffractometer
Absorption correction: multi-scan (*SADABS*; Sheldrick, 1996 ▶) *T*_min_ = 0.684, *T*_max_ = 0.746
5837 measured reflections
2099 independent reflections
1852 reflections with *I* > 2σ(*I*)
*R*_int_ = 0.022

#### Refinement

*R*[*F* ^2^ > 2σ(*F* ^2^)] = 0.040
*wR*(*F* ^2^) = 0.130
*S* = 1.08
2099 reflections
151 parameters
2 restraints
H atoms treated by a mixture of independent and constrained refinement
Δρ_max_ = 0.30 e Å^−3^
Δρ_min_ = −0.33 e Å^−3^

### 

Data collection: *APEX2* (Bruker, 2009 ▶); cell refinement: *SAINT* (Bruker, 2009 ▶); data reduction: *SAINT*; program(s) used to solve structure: *SHELXS97* (Sheldrick, 2008 ▶); program(s) used to refine structure: *SHELXL97* (Sheldrick, 2008 ▶); molecular graphics: *ORTEP-3* (Farrugia, 1997 ▶) and *DIAMOND* (Brandenburg, 2006 ▶); software used to prepare material for publication: *PLATON* (Spek, 2009 ▶) and *publCIF* (Westrip, 2010 ▶).

## Supplementary Material

Crystal structure: contains datablock(s) global, I. DOI: 10.1107/S1600536811022409/hb5906sup1.cif

Structure factors: contains datablock(s) I. DOI: 10.1107/S1600536811022409/hb5906Isup2.hkl

Supplementary material file. DOI: 10.1107/S1600536811022409/hb5906Isup3.cml

Additional supplementary materials:  crystallographic information; 3D view; checkCIF report

## Figures and Tables

**Table 1:** Hydrogen-bond geometry (Å, °)

*D*—H⋯*A*	*D*—H	H⋯*A*	*D*⋯*A*	*D*—H⋯*A*
O1—H1*o*⋯O2^i^	0.85 (1)	1.77 (2)	2.6119 (14)	173 (2)
O3—H3*o*⋯N3^ii^	0.85 (1)	1.96 (1)	2.7995 (16)	169 (2)
C6—H6⋯O3^iii^	0.95	2.60	3.5309 (19)	167
C8—H8⋯O3^iii^	0.95	2.23	3.1262 (18)	158

Symmetry codes: (i) 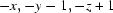; (ii) 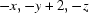; (iii) 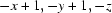.

**Fig. 1.**
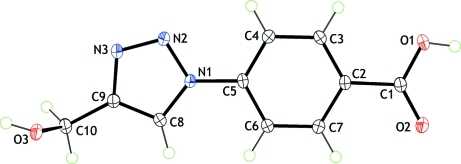
The molecular structure of compound (I) showing displacement ellipsoids at the 50% probability level.

**Fig. 2.**
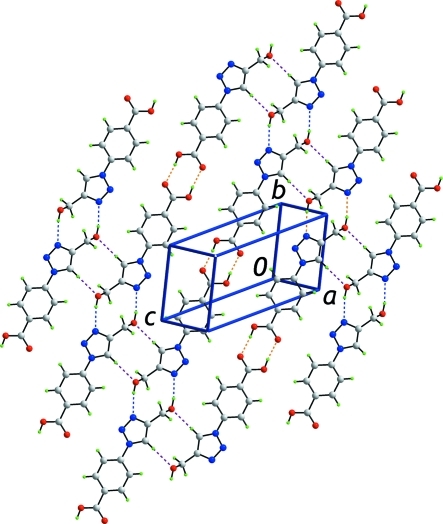
A view of the supramolecular layer in (I) mediated by O—H···O, O—H···N and C—H···O interactions, shown as orange, blue and purple dashed lines, respectively.

## supplementary crystallographic information

### Comment

The title compound, (I), is a precursor for the synthesis of fluorescent surfactants (McCaroll & Wandruzska, 1997). With the exception of the hydroxy substituent, the molecule of (I), Fig. 1, is essentially planar. The triazole ring is slightly twisted out of the plane of the benzene ring as seen in the value of the N2—N1—C5—C4 torsion angle of -6.43 (19) °; the dihedral angle between the rings is 6.32 (7) °. The carboxylic acid group is co-planar with the benzene ring to which it is attached: the O1—C1—C2—C3 torsion angle is 1.49 (19) °. The hydroxy group occupies a position almost perpendicular to the rest of the molecule with the N3—C9—C10—O3 torsion angle being -75.46 (16) °. Within the triazole ring, the sequence of N1—N2 [1.3562 (15) Å], N2—N3 [1.3141 (16) Å], N1—C8 [1.3579 (17) Å], N3—C9 [1.3627 (17) Å] and C8—C9 [1.3624 (19) Å] bond distances indicates considerable delocalization of π-electron density within the ring.

The crystal packing is dominated by O—H···O,*N* hydrogen bonding, Table 1. The carboxylic acid residues self-associate *via* the familiar eight-membered {··· HOC(═O)}_2_ synthon, and the hydroxy groups forms a hydrogen bond with the triazole-N3 atom *via* centrosymmetric 10-membered {···HOC_2_N}_2_ synthons. The result is the formation of a linear supramolecular chain with base vector [0 3 1], Fig. 2. Chains are connected into flat arrays *via* C—H···O hydrogen bonds and centrosymmetric ten-membered {···HC_3_O}_2_ synthons, Table 1 and Fig. 2. The closest interactions between layers are weak π···π contacts occurring between translationally related benzene and triazole rings [3.9433 (9) Å for symmetry operation *x*, 1 + *y*, *z*].

### Experimental

The title compound was synthesized *via* a Cu(I)-catalyzed cycloaddition between 4-azidobenzoic acid and propargyl alcohol after literature procedures (Rostovtsev *et al.*, 2002; Ryu & Zhao, 2005). 4-Azidobenzoic acid (1.0 g, 6.1 mmol) and propargyl alcohol (1.03 g, 18.4 mmol) were dissolved in methanol (20 ml) while stirring in the dark. Freshly prepared catalyst was prepared from the reduction of copper(II) sulfate (0.2 g, 0.08 mmol) with sodium ascorbate (0.4 g, 2 mmol) in about 2 ml of water (Himo *et al.*, 2005). The catalyst was then added into the mixture followed by stirring for 3 h. The crude product was dissolved in diethyl ether and washed with cold distilled water (50 ml). The organic layer was dried over magnesium sulfate, and the solvent was removed under vacuum to obtain 0.12 g (21%) of pure product. Yellow blocks were grown from its solution of THF (with a drop of ethyl acetate); *M*.pt. 529 - 532 K.

### Refinement

Carbon-bound H-atoms were placed in calculated positions (C—H 0.95 to 0.99 Å) and were included in the refinement in the riding model approximation, with *U_iso_*(H) = 1.2*U_eq_*(C). The O-bound H atoms were located in a difference map and their positions refined with *U_iso_*(H) = 1.5*U*_eq_(O).

### Crystal data

**Table:**

C_10_H_9_N_3_O_3_	*Z* = 2
*M**_r_* = 219.20	*F*(000) = 228
Triclinic, *P*1	*D*_x_ = 1.574 Mg m^−^^3^
Hall symbol: -P 1	Mo *K*α radiation, λ = 0.71073 Å
*a* = 5.4641 (7) Å	Cell parameters from 3318 reflections
*b* = 6.6596 (8) Å	θ = 3.1–30.7°
*c* = 13.1898 (16) Å	µ = 0.12 mm^−^^1^
α = 88.828 (2)°	*T* = 100 K
β = 83.577 (2)°	Block, yellow
γ = 75.828 (2)°	0.20 × 0.20 × 0.18 mm
*V* = 462.42 (10) Å^3^	

### Data collection

**Table:**

Bruker SMART APEX CCD diffractometer	2099 independent reflections
Radiation source: fine-focus sealed tube	1852 reflections with *I* > 2σ(*I*)
graphite	*R*_int_ = 0.022
ω scans	θ_max_ = 27.5°, θ_min_ = 1.6°
Absorption correction: multi-scan (*SADABS*; Sheldrick, 1996)	*h* = −7→7
*T*_min_ = 0.684, *T*_max_ = 0.746	*k* = −8→8
5837 measured reflections	*l* = −17→17

### Refinement

**Table:**

Refinement on *F*^2^	Primary atom site location: structure-invariant direct methods
Least-squares matrix: full	Secondary atom site location: difference Fourier map
*R*[*F*^2^ > 2σ(*F*^2^)] = 0.040	Hydrogen site location: inferred from neighbouring sites
*wR*(*F*^2^) = 0.130	H atoms treated by a mixture of independent and constrained refinement
*S* = 1.08	*w* = 1/[σ^2^(*F*_o_^2^) + (0.0804*P*)^2^ + 0.1593*P*] where *P* = (*F*_o_^2^ + 2*F*_c_^2^)/3
2099 reflections	(Δ/σ)_max_ < 0.001
151 parameters	Δρ_max_ = 0.30 e Å^−^^3^
2 restraints	Δρ_min_ = −0.33 e Å^−^^3^

### Special details

**Table:**

Geometry. All s.u.'s (except the s.u. in the dihedral angle between two l.s. planes) are estimated using the full covariance matrix. The cell s.u.'s are taken into account individually in the estimation of s.u.'s in distances, angles and torsion angles; correlations between s.u.'s in cell parameters are only used when they are defined by crystal symmetry. An approximate (isotropic) treatment of cell s.u.'s is used for estimating s.u.'s involving l.s. planes.
Refinement. Refinement of *F*^2^ against ALL reflections. The weighted *R*-factor *wR* and goodness of fit *S* are based on *F*^2^, conventional *R*-factors *R* are based on *F*, with *F* set to zero for negative *F*^2^. The threshold expression of *F*^2^ > 2σ(*F*^2^) is used only for calculating *R*-factors(gt) *etc*. and is not relevant to the choice of reflections for refinement. *R*-factors based on *F*^2^ are statistically about twice as large as those based on *F*, and *R*- factors based on ALL data will be even larger.

### Fractional atomic coordinates and isotropic or equivalent isotropic displacement parameters (Å^2^)

**Table:**

	*x*	*y*	*z*	*U*_iso_*/*U*_eq_	
O1	−0.21174 (19)	−0.26084 (15)	0.47377 (8)	0.0193 (3)	
H1O	−0.202 (4)	−0.376 (2)	0.5038 (14)	0.029*	
O2	0.19712 (19)	−0.40260 (15)	0.41945 (7)	0.0187 (3)	
O3	0.24825 (19)	0.81745 (15)	−0.07164 (7)	0.0188 (3)	
H3O	0.219 (4)	0.9413 (17)	−0.0937 (14)	0.028*	
N1	0.0479 (2)	0.47484 (17)	0.19947 (8)	0.0133 (3)	
N2	−0.1613 (2)	0.63139 (18)	0.19229 (9)	0.0163 (3)	
N3	−0.0881 (2)	0.76979 (18)	0.13169 (9)	0.0161 (3)	
C1	0.0044 (3)	−0.2560 (2)	0.42301 (10)	0.0141 (3)	
C2	0.0120 (2)	−0.06148 (19)	0.36720 (10)	0.0133 (3)	
C3	−0.2008 (3)	0.1064 (2)	0.37192 (10)	0.0144 (3)	
H3	−0.3530	0.0977	0.4123	0.017*	
C4	−0.1908 (3)	0.2860 (2)	0.31786 (10)	0.0149 (3)	
H4	−0.3345	0.4007	0.3214	0.018*	
C5	0.0336 (2)	0.2951 (2)	0.25830 (10)	0.0131 (3)	
C6	0.2481 (3)	0.1299 (2)	0.25451 (10)	0.0145 (3)	
H6	0.4008	0.1394	0.2146	0.017*	
C7	0.2373 (3)	−0.0480 (2)	0.30923 (10)	0.0148 (3)	
H7	0.3831	−0.1608	0.3073	0.018*	
C8	0.2538 (3)	0.5157 (2)	0.14328 (10)	0.0155 (3)	
H8	0.4227	0.4318	0.1356	0.019*	
C9	0.1648 (2)	0.7034 (2)	0.10030 (10)	0.0146 (3)	
C10	0.3059 (3)	0.8278 (2)	0.03094 (10)	0.0171 (3)	
H10A	0.2585	0.9739	0.0543	0.021*	
H10B	0.4908	0.7740	0.0335	0.021*	

### Atomic displacement parameters (Å^2^)

**Table:**

	*U*^11^	*U*^22^	*U*^33^	*U*^12^	*U*^13^	*U*^23^
O1	0.0197 (5)	0.0161 (5)	0.0217 (5)	−0.0063 (4)	0.0026 (4)	0.0063 (4)
O2	0.0211 (5)	0.0135 (5)	0.0195 (5)	−0.0024 (4)	0.0010 (4)	0.0038 (4)
O3	0.0226 (5)	0.0145 (5)	0.0167 (5)	−0.0011 (4)	−0.0003 (4)	0.0049 (4)
N1	0.0136 (5)	0.0111 (5)	0.0149 (5)	−0.0028 (4)	−0.0013 (4)	0.0033 (4)
N2	0.0152 (6)	0.0136 (6)	0.0188 (6)	−0.0019 (4)	−0.0010 (4)	0.0050 (4)
N3	0.0167 (6)	0.0138 (5)	0.0175 (6)	−0.0039 (4)	−0.0008 (4)	0.0050 (4)
C1	0.0170 (6)	0.0132 (6)	0.0128 (6)	−0.0054 (5)	−0.0012 (5)	0.0011 (5)
C2	0.0162 (7)	0.0116 (6)	0.0129 (6)	−0.0050 (5)	−0.0017 (5)	0.0017 (5)
C3	0.0139 (6)	0.0150 (6)	0.0148 (6)	−0.0054 (5)	0.0003 (4)	0.0021 (5)
C4	0.0135 (6)	0.0137 (6)	0.0166 (6)	−0.0019 (5)	−0.0014 (5)	0.0025 (5)
C5	0.0159 (6)	0.0121 (6)	0.0124 (6)	−0.0056 (5)	−0.0020 (5)	0.0029 (5)
C6	0.0138 (6)	0.0139 (6)	0.0155 (6)	−0.0039 (5)	0.0001 (5)	0.0025 (5)
C7	0.0154 (6)	0.0123 (6)	0.0159 (6)	−0.0026 (5)	−0.0009 (5)	0.0018 (5)
C8	0.0142 (6)	0.0153 (6)	0.0165 (6)	−0.0042 (5)	0.0007 (5)	0.0031 (5)
C9	0.0148 (6)	0.0137 (6)	0.0154 (6)	−0.0039 (5)	−0.0016 (5)	0.0021 (5)
C10	0.0175 (6)	0.0166 (6)	0.0173 (7)	−0.0052 (5)	−0.0009 (5)	0.0048 (5)

### Geometric parameters (Å, °)

**Table:**

O1—C1	1.2982 (16)	C3—C4	1.3891 (18)
O1—H1O	0.848 (9)	C3—H3	0.9500
O2—C1	1.2456 (17)	C4—C5	1.3939 (18)
O3—C10	1.4299 (17)	C4—H4	0.9500
O3—H3O	0.852 (9)	C5—C6	1.3944 (18)
N1—N2	1.3562 (15)	C6—C7	1.3852 (18)
N1—C8	1.3579 (17)	C6—H6	0.9500
N1—C5	1.4266 (16)	C7—H7	0.9500
N2—N3	1.3141 (16)	C8—C9	1.3624 (19)
N3—C9	1.3627 (17)	C8—H8	0.9500
C1—C2	1.4838 (18)	C9—C10	1.4970 (18)
C2—C7	1.3961 (18)	C10—H10A	0.9900
C2—C3	1.3985 (18)	C10—H10B	0.9900
			
C1—O1—H1O	110.8 (14)	C4—C5—N1	120.37 (12)
C10—O3—H3O	106.1 (13)	C6—C5—N1	118.51 (12)
N2—N1—C8	110.82 (11)	C7—C6—C5	119.59 (12)
N2—N1—C5	121.00 (11)	C7—C6—H6	120.2
C8—N1—C5	128.16 (11)	C5—C6—H6	120.2
N3—N2—N1	106.38 (11)	C6—C7—C2	119.94 (12)
N2—N3—C9	109.65 (11)	C6—C7—H7	120.0
O2—C1—O1	123.72 (12)	C2—C7—H7	120.0
O2—C1—C2	120.44 (12)	N1—C8—C9	104.78 (12)
O1—C1—C2	115.84 (12)	N1—C8—H8	127.6
C7—C2—C3	120.00 (12)	C9—C8—H8	127.6
C7—C2—C1	118.69 (12)	C8—C9—N3	108.36 (11)
C3—C2—C1	121.31 (12)	C8—C9—C10	129.04 (12)
C4—C3—C2	120.37 (12)	N3—C9—C10	122.59 (12)
C4—C3—H3	119.8	O3—C10—C9	110.65 (11)
C2—C3—H3	119.8	O3—C10—H10A	109.5
C3—C4—C5	118.94 (12)	C9—C10—H10A	109.5
C3—C4—H4	120.5	O3—C10—H10B	109.5
C5—C4—H4	120.5	C9—C10—H10B	109.5
C4—C5—C6	121.12 (12)	H10A—C10—H10B	108.1
			
C8—N1—N2—N3	0.21 (15)	C8—N1—C5—C6	−5.0 (2)
C5—N1—N2—N3	−178.52 (11)	C4—C5—C6—C7	1.2 (2)
N1—N2—N3—C9	−0.04 (15)	N1—C5—C6—C7	−178.62 (11)
O2—C1—C2—C7	1.3 (2)	C5—C6—C7—C2	0.4 (2)
O1—C1—C2—C7	−178.57 (11)	C3—C2—C7—C6	−1.5 (2)
O2—C1—C2—C3	−178.67 (12)	C1—C2—C7—C6	178.55 (12)
O1—C1—C2—C3	1.49 (19)	N2—N1—C8—C9	−0.29 (15)
C7—C2—C3—C4	1.0 (2)	C5—N1—C8—C9	178.32 (12)
C1—C2—C3—C4	−179.03 (11)	N1—C8—C9—N3	0.26 (15)
C2—C3—C4—C5	0.6 (2)	N1—C8—C9—C10	179.62 (13)
C3—C4—C5—C6	−1.7 (2)	N2—N3—C9—C8	−0.14 (16)
C3—C4—C5—N1	178.15 (11)	N2—N3—C9—C10	−179.55 (12)
N2—N1—C5—C4	−6.43 (19)	C8—C9—C10—O3	105.27 (16)
C8—N1—C5—C4	175.08 (12)	N3—C9—C10—O3	−75.46 (16)
N2—N1—C5—C6	173.44 (11)		

### Hydrogen-bond geometry (Å, °)

**Table:**

*D*—H···*A*	*D*—H	H···*A*	*D*···*A*	*D*—H···*A*
O1—H1O···O2^i^	0.85 (1)	1.77 (2)	2.6119 (14)	173.(2)
O3—H3O···N3^ii^	0.85 (1)	1.96 (1)	2.7995 (16)	169 (2)
C6—H6···O3^iii^	0.95	2.60	3.5309 (19)	167
C8—H8···O3^iii^	0.95	2.23	3.1262 (18)	158

Symmetry codes: (i) −*x*, −*y*−1, −*z*+1; (ii) −*x*, −*y*+2, −*z*; (iii) −*x*+1, −*y*+1, −*z*.
